# Biventricular surgical repair of “Swiss Cheese” ventricular septal defects with two-patch and right ventricle apex excluding technique: preliminary experience and clinical results

**DOI:** 10.1186/s13019-021-01399-w

**Published:** 2021-03-20

**Authors:** Qin Wu, Lei Shi, Rui Chen, Quansheng Xing

**Affiliations:** 1 Heart Center, Qingdao Women and Children’s Hospital, Qingdao University, No.6 Tongfu Road, 266034 Qingdao, People’s Republic of China; 2 Pediatric Echocardiography Lab, Qingdao Women and Children’s Hospital, Qingdao University, No.6 Tongfu Road, 266034 Qingdao, People’s Republic of China

**Keywords:** “Swiss cheese” ventricular septal defects, Congenital heart disease, Surgical repair

## Abstract

**Background:**

**“**Swiss Cheese**”** ventricular septal defects (VSDs) is a kind of rare and complex congenital heart defects and the surgical management remains controversial and a challenge. We reviewed our preliminary clinical experience on biventricular surgical repair of “Swiss Cheese” VSDs with two-patch and right ventricle apex excluding technique in 10 cases.

**Methods:**

From May 2014 to December 2019, a series of 10 patients (M/F = 3/7) were admitted in our center. Nine cases underwent one-stage surgical repair with two-patch and right ventricle apex excluding technique and 1 case received two-stage surgical repair with the same technique. Surgical repair was done with cardiopulmonary bypass (CPB) in all cases. Two fresh autologous pericardium patches were used to close defects of the outflow tract area and the apex trabecular area respectively and as a result, the right ventricular apex was excluded from the right ventricular inflow tract.

**Results:**

All operations were successful. Median CPB time and aortic clamping time were 96 min and 68 min respectively. Delayed chest closure was performed in 2 cases within 48–72 h postoperatively. The Median time of mechanical ventilation and ICU stay were 131.3 h and 8 days respectively. Median length of hospital stay after operation was 11 (9–42) days. There was no mortality and major complication except for 2 cases of ventilator associated pneumonia. There was no death and major complication during a median follow-up time of 3.2 years.. The latest echocardiography results showed the left and right heart function was normal in all the cases.

**Conclusions:**

Biventricular surgical repair of “Swiss Cheese” VSDs with two-patch of fresh autologous pericardium and right ventricle apex excluding technique in infants is safe and feasible with favorable early and mid-term results. Long term results need to be evaluated with more cases.

## Background

“Swiss cheese” ventricular septal defects (VSDs) is a rare and most serious form of multiple VSDs, which was defined as 4 or more muscular VSDs by the Congenital Heart Surgery Nomenclature [1]. Patients with “Swiss cheese” VSDs always need surgical treatment in their very early stage after birth because of severe cardiac dysfunction. To do a one-stage repair of “Swiss cheese” VSDs in infants is technically challenging because of the difficulty in visualizing, accessing and closing every “hole” as these kind of VSDs invariably involves all components of the ventricular septum and mostly consists of uncountable big and small VSDs. Treatment options include palliative pulmonary banding, primary surgical repair with a right or left or both right and left ventriculotomies, hybrid transcatheter or intraoperative device closure, sandwich technique and re-endocardialization strategy. Despite of the improvements in repairing this challenging pathology, the overall results are still barely satisfactory with significant operative mortality and complications, including ventricular dysfunction, residual shunt and complete heart block [2–5].

In the past 5 years, we have developed a new treatment strategy using right ventricle apex excluding technique with two fresh autologous pericardium patches and applied this strategy in 10 patients with satisfactory results. The aim of this strategy was to close the rim of two divided areas of the VSDs associated ventricular septum and avoid reducing right ventricular size and impairing right ventricular diastolic function in the long run. In this report, we discuss our experiences in performing the technique and the preliminary results.

## Methods

### Patients

Since May 2014, 10 consecutive infants were referred to our institution for “Swiss cheese” VSDs and 3 of them had major combined cardiac lesions. One patients had previously undergone a palliative procedure of main pulmonary artery banding in his neonatal period due to uncontrollable heart failure. The patients’ general characteristics are listed in Table 1. This study was approved by the institutional review board of the ethics committee in our hospital (QFELL-KY-2020-07).

**Table 1:** Preoperative Characteristics of the 10 patients

Gender(F/M)	7/3
Age (Mo), median (range)	5 (2–13)
Weight (Kg)	4.5 (3.7–7.0)
Associated cardiac lesions	
Tetralogy of Fallot	1
Double-outlet right ventricle	1
Coarctation of the Aorta	1
Perimembranous VSD	2
Atrial septal defect	3
Patent ductus arteriosis	1
Cardio-thoracic ratio	0.64 (0.58–0.72)
ECG	
iRBBB/cRBBB	3
iLBBB	1
Previous cardiac interventions	
Main pulmonary artery banding	1

*ECG* electrocardiogram, *iRBBB* incomplete right bundle branch block, *cRBBB* complete or complete right bundle branch block, *iLBBB* incomplete left bundle branch block

### Procedure

The operation was performed through a median sternotomy with mild hypothermic (32–34 °C) cardiopulmonary bypass (CPB) established by standard aortic and bicavalcannulation. Myocardial protection was achieved by using one single dose of antegrade cold (4 °C–8 °C) HTK cardioplegic solution after aortic cross-clamping. A longitudinal right atriotomy was done and stay sutures were placed on the inner wall of the right atrium to evert the tricuspid valve.

**Fig. 1:**
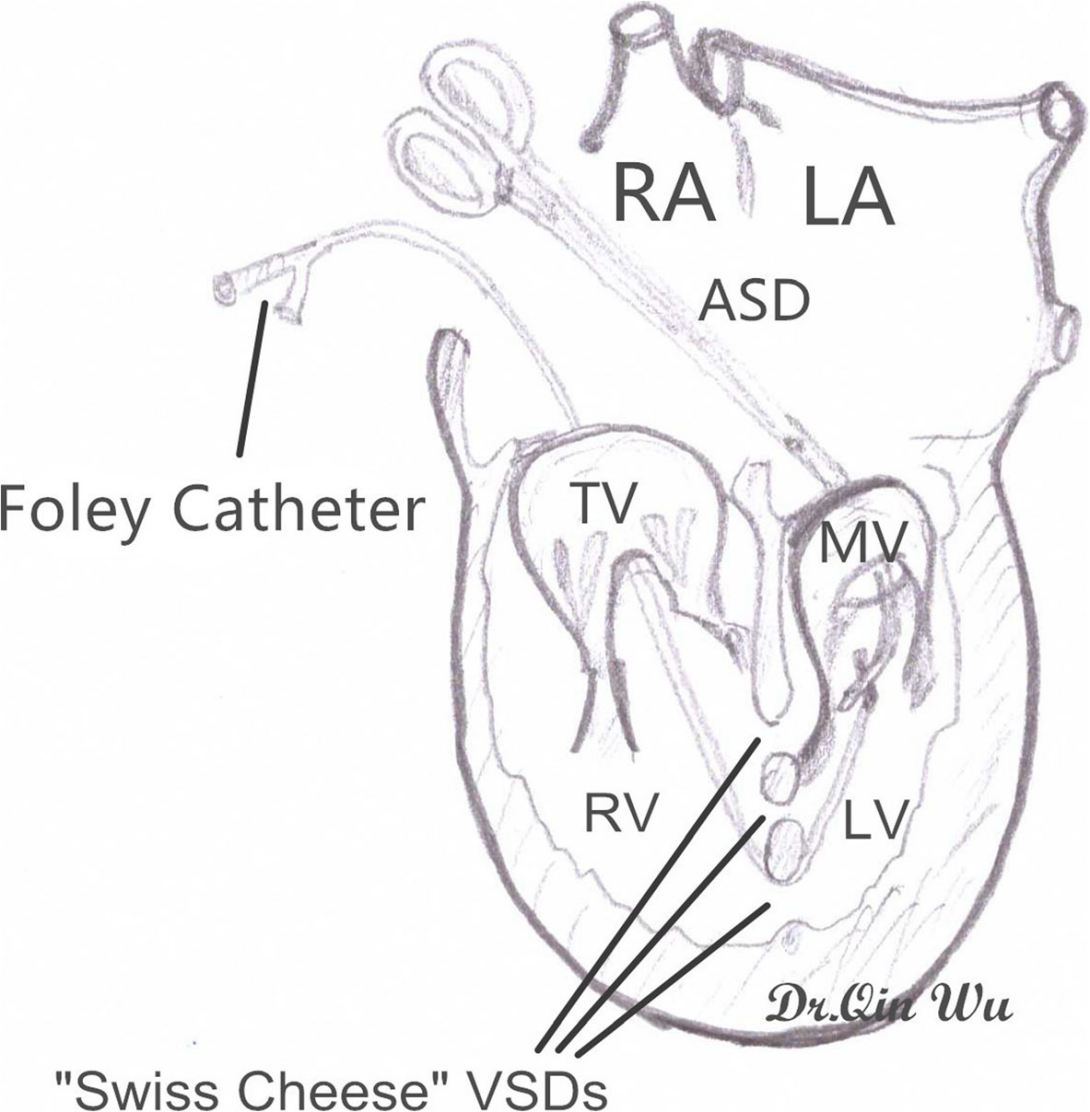
Schematic picture illustrating how to get a better visualization of the “Swiss Cheese” VSDs with the help of a Foley Catheter. The interventricular septum was exposed through the TV orifice and a right angle clamp was passed through ASD, MV orifice, LV cavity and then one big “hole” of the “Swiss Cheese” VSDs to the RV cavity. A Foley Catheter was caught and withdrew to meet the other end of the catheter. With gently pulling tension of the Foley catheter, the whole rim of the “Swiss Cheese” VSDs can be clearly visualized RA: right atrium; LA: left atrium; ASD: atrial septal defect; TV: tricuspid valve; MV: mitral valve; RV: right ventricle; LV: left ventricle

**Fig. 2:**
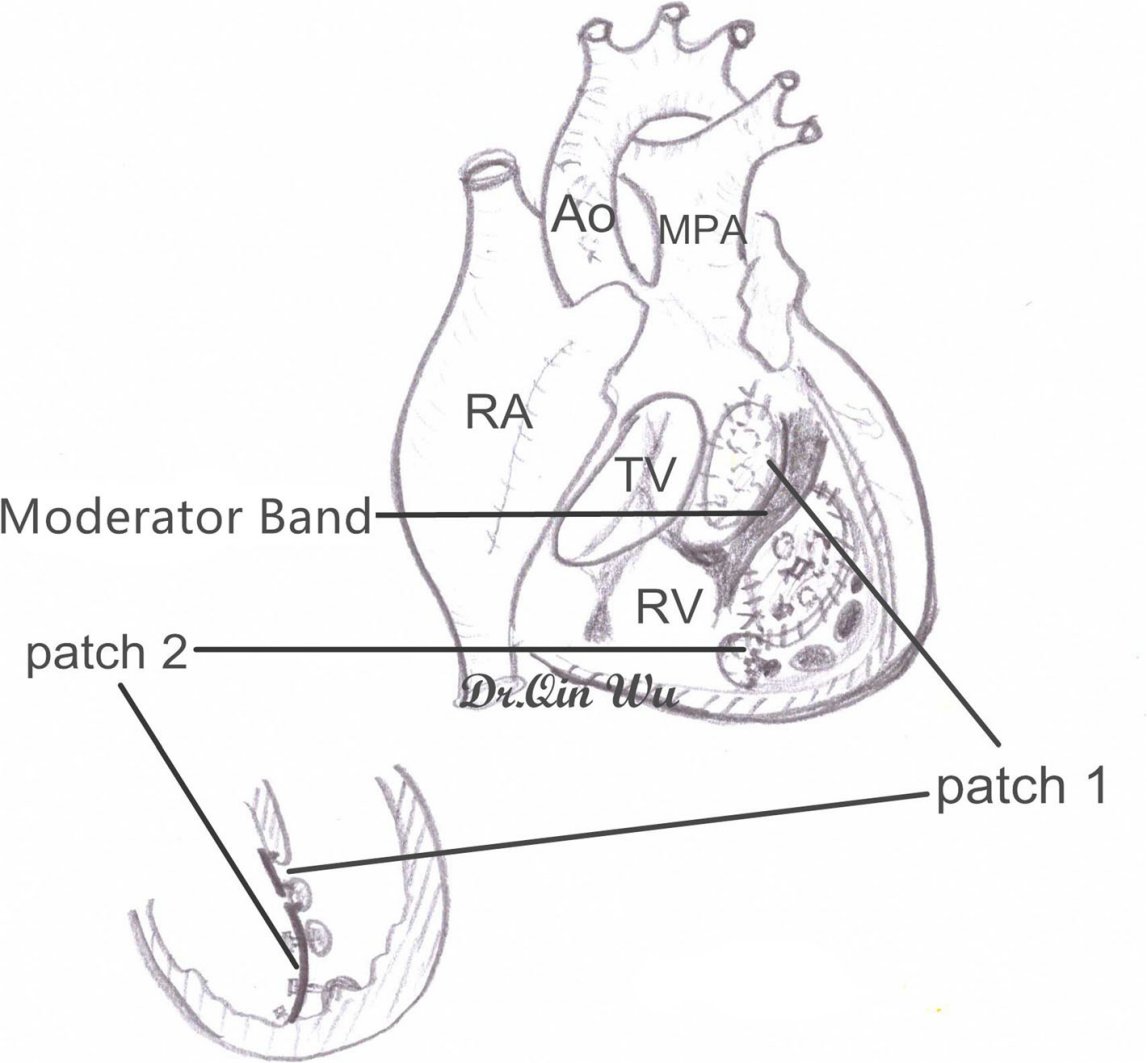
Schematic picture illustrating the result of closing the “Swiss Cheese” VSDs with two patches of fresh pericardium and excluding the right ventricular apex**.** The “Swiss Cheese” VSDs could be always divided into two portions, the outflow tract area defects and the apex trabecular area defects by the moderator band. Two fresh autologous pericardium patches of corresponding size were sutured to cover the two portions including all the VSDs. During suturing the lower patch, 3 to 5 6–0 or 5–0 polypropylene interrupted pledgetted sutures were evenly placed between the patch and the trabecular muscles Ao: aorta; MPA: main pulmonary artery; RA: right atrium; TV: tricuspid valve; RV: right ventricle

**Fig. 3:**
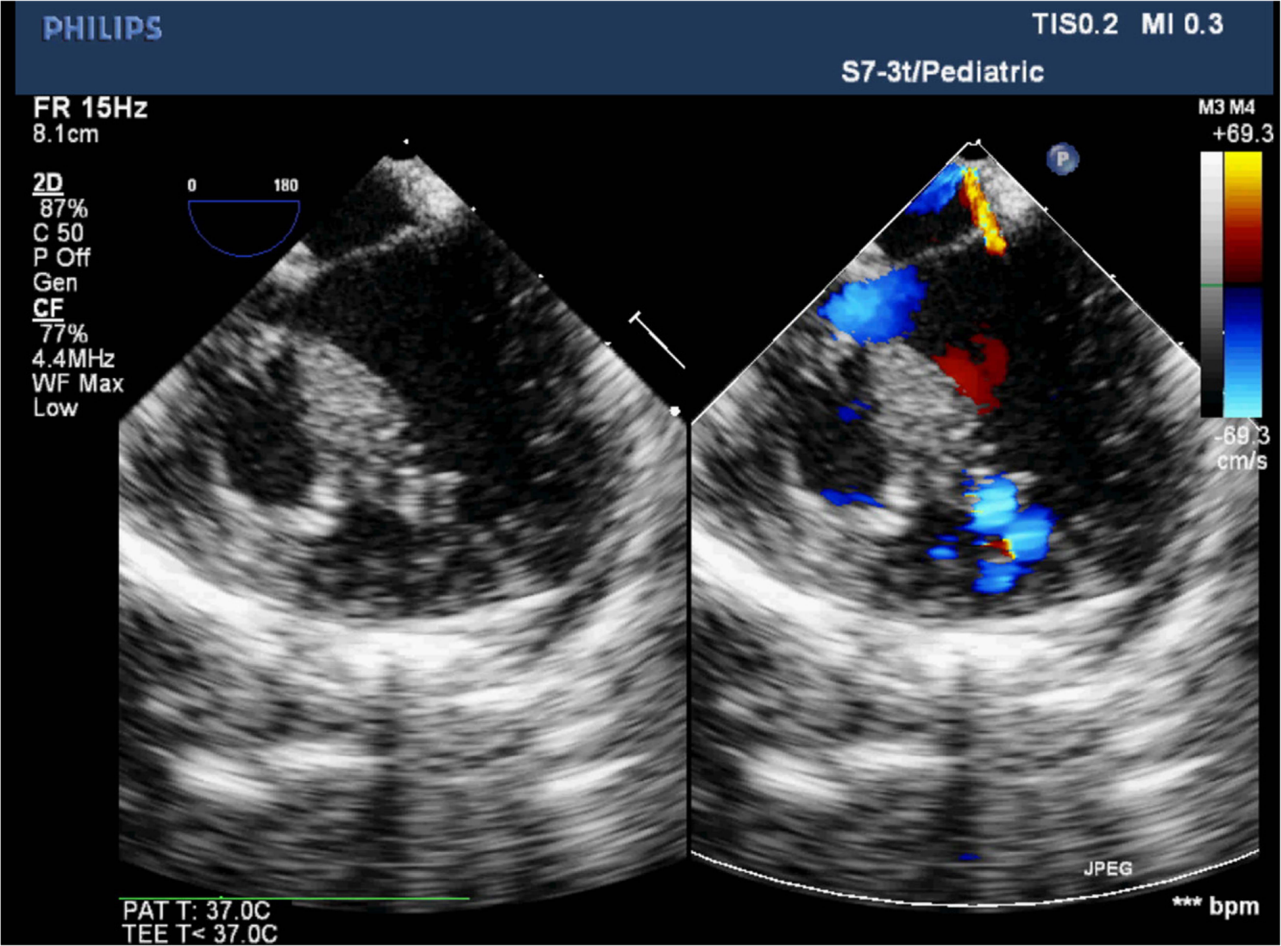
Transesophageal echocardiography (four chamber view) picture showing the apical portion of the right ventricle is excluded from the right ventricle and all the left-to right ventricular shunts are obliterated, although there are still blood flows inside the interventricular septum

The interventricular septum was exposed through the tricuspid valve orifice and a right angle clamp was passed through the atrial septum defect (ASD) or surgically created ASD, mitral valve orifice, left ventricular cavity and then one big “hole” of the “Swiss Cheese” VSDs to the right ventricular cavity. A 10 French size silicone Foley catheter was caught and withdrew to pass through the big “hole”, mitral valve orifice and ASD to meet the other end of the catheter by the right angle clamp. With gently pulling tension of the Foley catheter, the whole rim of the “Swiss Cheese” VSDs can be clearly visualized (Fig. 1). The “Swiss Cheese” VSDs involving interventricular septum could be always divided into two portions, the outflow tract area defects and the apex trabecular area defects, in the surgeon’s mind by the moderator band. The sizes of the two areas were measured and two fresh autologous pericardium patches of corresponding size were harvested and sutured along the edge of the portion with 6–0 or 5–0 polypropylene running suture respectively (Fig. 2). Sometimes, the upper patch would be sutured at the edge of the moderator band to make sure there was no leakage underneath. The lower patch was always sutured between the moderator band and the anterior wall of the right ventricular to exclude the apical portion of the right ventricle and obliterate all the left to right ventricular shunts. During suturing the lower patch, 3 to 5 6–0 or 5–0 polypropylene interrupted pledgetted sutures were evenly placed between the patch and the trabecular muscles to avoid septal bulging and prevent diminution of the right ventricular cavity or inflow occlusion across the inlet septum (Fig. 2). Combined cardiac lesions were corrected during the procedure. The tricuspid valve was tested for functional competence by infusion of saline into the right ventricle. Pacing wires were placed in all the cases and transesophageal echocardiography (TEE) was routinely done to evaluate the surgical results during the rewarming period (Fig. 3). Patient was wean off the CPB after TEE test. A mediastinal drainage tube was set and sternum was closed. Delayed chest closure was carried out to prevent cardiac tamponade in some cases.

### Postoperative treatment and follow-up

Postoperative pulmonary hypertension was managed with phosphodiesterase inhibitor (Milrinone) in all the patients and treprostinil (Remodulin) in 3 cases with severe pulmonary hypertension. Chest radiography, electrocardiography (ECG), and TTE were performed before discharge. Outpatient follow-up was with clinical examination, ECG and TTE at 1 month, 3 months, 6 months, 1 year, and then yearly.

## Results

All the operations were successful. Median CPB time and aortic clamping time were 96(68–167) min and 68(43–122) min respectively. The median time for exposure and closure of “Swiss Cheese” VSDs was 56 (38–75) min. TEE test in the operating room showed that there were 2 cases with less than minor residual shunts (case 1 φ = 1.0 mm & flow velocity = 2.1 m/s; case 2 φ = 1.2 mm & flow velocity = 2.6 m/s), no more than mild tricuspid regurgitation, no septal bulging induced right ventricular outflow obstruction and cardiac dysfunction. ECG showed sinus rhythm in all patients, with right bundle block in 5 cases. Delayed chest closure were done in 2 cases within 48–72 h postoperatively. The time of mechanical ventilation and ICU stay were 131.3(32–328) hours and 8(5–31) days respectively. Median length of hospital stay after operation was 11 (9–42) days. There was no mortality and major complication except for 2 cases of ventilator associated pneumonia. TTE test before discharge room showed that residual shunt remain the same in case 2 (φ = 1.2 mm & flow velocity = 2.3 m/s) and closed in case 1. No heart block was detected in ECG in any of the patients.

All the patients were followed up clinically and by echocardiography for 3.2 years (3 month-5.5 years). There was no death, major complication and reoperation. All the patients were asymptomatic in sinus rhythm and without medication. The latest TTE results showed that the left and right heart volume and function were normal in all the cases, without any residual shunt.

## Discussion

VSDs are the most common congenital heart disease (CHD), making up about 20 to 30% as an isolated lesion or more than 50% in conjunction with other cardiac anomalies of all the congenital cardiac lesions. Muscular VSDs, the type 4 of VSDs, is relatively uncommon and accounting for 5–12% of all the VSDs [6]. “Swiss Cheese” VSDs is a subtype of muscular VSDs and most serious form, which is the result of non-contraction of ventricular septum during embryonic development [7]. As conventional surgical repair of “Swiss cheese” VSDs is generally accompanied by substantial early and late mortality and morbidity (8.5–25%) [8], much higher than that of other VSDs, it is important to treat “Swiss cheese” VSDs as one kind of very complex CHDs but not common VSDs and should attract much more attention of the pediatricians.

Historically, pulmonary artery banding was the preferred treatment of “Swiss cheese” VSDs in most of the patients. The aim is to protect the pulmonary vascular bed and relieves heart failure and win more time for further treatment, biventricular repair or palliative cavopulmonary connection [6, 9]. However, as pulmonary artery banding may lead to various complications afterwards, such as right ventricular hypertrophy, right ventricular outflow tract obstruction distortion of the pulmonary artery and inadequate protection of the pulmonary vascular bed, this technique may be only appropriate in selected patients who are sufficiently ill and cannot reasonably be expected to tolerate CPB.

With improvement of surgical techniques and perioperative management in the past several decades, a growing number of surgeons prefered single-stage repair but developed different opinions and surgical techniques including direct closure through ventriculotomy, sandwich patch technique or hybrid closure. However, each author has been successful with his/her technique, yet no technique is uniformly reproducible. Short and long term complications are still the cornerstones of the surgical results [10], and pediatric cardiac surgeons are still challenged by the tough treatments of “Swiss cheese” VSDs [11]. Therefore, they have been seeking more ideal techniques for the treatment of this complex VSD. In 2006, Alsoufi and his colleagues [12] reported a new approach, the transatrial re-endocardialization of interventricular septum, to treat “Swiss cheese” VSDs. Although the outcome has been improved with low incidence of permanent heart block, this technique has not been widely used because of its technical difficulty and long CPB and procedure time.

The technique of using a large patch extending on to the right ventricular free wall and exclusion of part of the right ventricular apex provided a simple solution in the last several decades. Macé L and his colleagues [13] modified this technique with intermediate fixings to avoid septal bulging induced cardiac dysfunction. They applied this modified single patch technique in 5 cases with promising short term results. However, long term in adult life, the reduced size of right ventricle, and diastolic dysfunction cause problem, such as cyanosis, right heart failure, cirrhosis, arrhythmia [14]. The reason may be related to the fact that in most of the cases, the material of the patches being used to close the defects were Dacron and did not have growth potential. Furthermore, the patch was too large and it might cover the entire endocardium of the right ventricle. These would not only limit the development of the right ventricle, but also lead to diffuse fibrosis and calcification of the right ventricular endocardium years later. Right ventricular dysfunction would be the results, and then the increasing pressure in the right heart, that could cause right atrium dilation, reopening of the foramen ovale induced cyanosis and arrhythmia.

In 2014, we developed a modified strategy to treat “Swiss cheese” VSDs to address the potential drawbacks of previously reported single patch technique but based on the same technical principle of exclusion of the right ventricular apex. We tried to use two fresh pericardium patches to close the defects during the procedure instead of a large prosthetic patch, like Darcon or Gore-tex. The main technical points were described above in the procedure part. Our technique is suitable for infants over 3 months old. Although the CPB time (96 min) and aortic clamping time (68 min) in our series of cases are longer than that of previous reported research [13], if we take a look at the median time for exposing and closing of “Swiss Cheese” VSDs, 56 min is relatively short and the advantages of our technique can be easily recognized in terms of technical requirements and operation time. However, it is pretty hard to apply this technique in patients under 3 months old as a full view exposure of the VSDs is almost impossible. If the patient’s condition is too critical to wait for receiving this technique till 3 months old, pulmonary artery banding can be performed in advance. In this group, one case was taken according to this strategy, and finally had a biventricular repair with good result. The fact that there were no serious complication in the early postoperative period in this study indicates that this technique is safe and reliable. There was no serious complication during a median follow-up period of 3.2 years. Follow-up TTE test showed normal systolic and diastolic cardiac function in all the 10 cases. This may be related to the use of fresh pericardial patches, as it was reported that fresh autologous pericardium showed good biological characteristics, such as free of retraction, thickening, stiffness, fibrosis and calcification [15], thus it possibly does not affect right ventricular function in midterm period. In addition, the two-patch technique not only protects the moderator band, an important anatomical structure of the right ventricle, but also avoids a too large patch to cover the entire inner right ventricular wall and thus limiting the diastolic function of the right ventricle.

## Conclusion

Biventricular surgical repair with two-patch of fresh autologous pericardium and right ventricle apex excluding technique is an optimal strategy in surgical treatment of “Swiss cheese” VSDs. This strategy has all of the advantages, including being easy to manipulate, secure closure of the defects, low incidence of severe arrhythmia and other complications. It can be applied in infant patents with promising early and mid-term results. But more experiences and long-term follow-up results are needed to provide further information regarding all these aspects.

## Data Availability

Please contact author for data requests.

## Abbreviations

VSDs: Ventricular septal defects
CPB: Cardiopulmonary bypass;
ASD: Atrial septum defect
TEE: Transesophageal echocardiography
ECG: Electrocardiography
CPB: Cardiopulmonary bypass
CHD: Congenital heart defects.
